# Current practice of concomitant surgeries in cartilage repair of the femorotibial compartment of the knee: baseline data of 4968 consecutive patients from the German cartilage registry (KnorpelRegister DGOU)

**DOI:** 10.1007/s00402-021-04077-7

**Published:** 2021-07-29

**Authors:** Johannes Zellner, Svea Faber, Gunter Spahn, Wolfgang Zinser, Philipp Niemeyer, Peter Angele

**Affiliations:** 1 Department of Trauma Surgery, University Medical Center of Regensburg, Franz Josef Strauss Allee 11, 93042 Regensburg, Germany; 2 Sporthopaedicum Regensburg, Hildegard von Bingen Strasse 1, 93053 Regensburg, Germany; 3 OCM, Orthopedic Surgery Munich, Steinerstrasse 6, 812306 Munich, Germany; 4 Praxisklinik Eisenach, Sophienstrasse 16, 99817 Eisenach, Germany; 5 Department of Trauma Surgery, University Medical Center Jena, Bachstrasse 18, 07743 Jena, Germany; 6 Department of Orthopedics and Trauma Surgery, St. Vinzenz Hospital, Dr.-Otto-Seidel-Strasse 31-33, 46535 Dinslaken, Germany; 7 Department of Orthopedics and Trauma Surgery, University Medical Center Freiburg, Hugstetter Strasse 55, 79106 Freiburg, Germany

**Keywords:** Cartilage treatment knee, Concomitant surgery, Comorbidity, Osteotomy, Instability, Meniscus

## Abstract

**Introduction:**

The treatment of underlying comorbidities is a field of rising interest in cartilage repair surgery. The aim of this study was to analyze the current practice of concomitant surgeries in cartilage repair of the knee especially in the medial or lateral femorotibial compartment. Type, frequency and distribution of additional surgeries for correction of malalignment, knee instability and meniscus deficiency should be evaluated.

**Methods:**

Baseline data of 4968 patients of the German Cartilage Registry (KnorpelRegister DGOU) were analyzed regarding the distribution of concomitant surgeries in addition to regenerative cartilage treatment.

**Results:**

Beyond 4968 patients 2445 patients with cartilage defects in the femorotibial compartment of the knee could be identified. Of these patients 1230 (50.3%) received additional surgeries for correction of malalignment, instability and meniscus deficiency. Predominant procedures were leg axis corrections (31.3%), partial meniscectomy (20.9%) and ACL reconstruction (13.4%). The distribution of the concomitant surgeries varied between cartilage defects according to the different defect genesis. Patients with traumatic defects were younger (36y) and received predominantly ACL reconstructions (29.2%) (degenerative: 6.7%), whereas patients with degenerative defects were older (43y) and underwent predominantly leg axis corrections (38.0%; traumatic: 11.0%).

**Conclusions:**

This study shows the high frequency and distinct distribution of the concomitant surgeries in addition to regenerative cartilage treatment procedures. Understanding of the underlying cause of the cartilage defect and addressing the comorbidities as a whole joint therapy are of utmost importance for a successful regenerative cartilage treatment. These data provide a baseline for further follow up evaluations and long-term outcome analysis.

**Level of evidence:**

II.

## Introduction

Articular cartilage injuries are common. They can result from acute traumatic injuries, posttraumatic or early degenerative changes, osteochondritis dissecans or avascular necrosis. Numerous reports analyzing high numbers of arthroscopies show cartilage lesions in up to 60% of the patients [1]. It is generally agreed that the persistence of cartilage defects is a risk factor for joint dysfunction, which finally may lead to osteoarthritis [2, 3].

Since decades regenerative treatment options for small and middle-sized cartilage lesions were developed like e.g. Pridie-drilling or microfracture. Large size cartilage defects can be successfully addressed by matrix-induced chondrocyte transplantation techniques [4–7].

However treatment of cartilage defects is still challenging with an overall failure rate of regenerative cartilage repair procedures of up to 25% [8, 9]. Various factors influence the regeneration potential of chondral lesions and the outcome of operative repair procedures. Such factors are patient-specific factors (e.g. age, weight, smoker status, activity level, inflammatory milieu), joint specific factors (e.g. meniscus status, malalignment, ligament instability, maltracking) and defect-specific factors (e.g. previous surgery, containment of the defect, subchondral bone quality, defect size, defect location, number of defects, age of defect) [9].

The different chondral defect conditions and the comorbidities of the knee joint should affect the surgeon´s decision-making process for choosing the appropriate treatment methods and algorithms for specific defect entities.

In their review Niemeyer et al. give recommendations for different cartilage treatment options according to certain defect-specific factors [10]. Based on current evidence, an indication for matrix-guided autologous chondrocyte transplantation (MACT) is given for symptomatic cartilage defects starting from defect sizes of more than 2.5 cm^2^. Smaller lesions are supposed to be treated by bone marrow stimulating techniques like microfracturing. In addition, the status of the subchondral bone should influence the decision-making process for cartilage therapy. Smaller osteochondral defects are best treated with autologous osteochondral transplantation (OCT). For large and deep osteochondral lesions, a combination of MACT and bone augmentation techniques is the favorable treatment option.

Recent studies show that the clinical outcome of cartilage repair strategies improves if patient- and joint specific factors are addressed in the treatment algorithm of cartilage defects besides defect-specific factors. Only if the comorbidities are addressed sufficiently, the chance for appropriate cartilage regeneration is achievable [11]. These results are based on small cohorts mainly treated in a single institution, which does not represent the treatment reality overall. Krych et al. analyzed the mode of failure of primary procedures for cartilage repair and detected that patients after microfracturing were more likely to have unsatisfactory results. Main reasons for failure of cartilage therapy were persisting and not addressed malalignment, meniscal deficiency and instability of the knee. Sheppard et al. found a substantial inconsistency in reporting clinical outcome associated with concomitant procedures relative to outcomes related to cartilage repair. In their review on knee cartilage restoration recognition and management of concomitant pathology is inadequately reported in approximately 28% of studies.

Register data might help to overcome these limitations. Models for the effectiveness of this type of data collection are the Scandinavian register for arthroplasty [12] or ACL register [13].

In 2013 the German Cartilage Registry (KnorpelRegister DGOU) was initiated to gather information about the treatment reality of cartilage lesions in daily clinical practice. In this study preliminary baseline data of the whole registry are presented regarding the cartilage treatment procedures in the femorotibial compartment and their concomitant surgeries with special focus on defect size and genesis of the cartilage defect.

## Methods

### Registry and data collection

Data for the present analysis have been evaluated and taken from the German Cartilage Registry (KnorpelRegister DGOU). The KnorpelRegister DGOU is an observational, nation-wide and longitudinal multi-center registry of patients assigned for surgical treatment for cartilage defects of the knee, and aims to determine real-life treatment patterns and clinical outcomes. The registry was initiated by the Working Group Clinical Tissue Regeneration of the German Society for Orthopedics and Trauma (DGOU) in 2013. The study design of the German Cartilage Registry was described in recent publications [14, 15]. Since then, the number of sites has increased up to 120. The registry is conducted in accordance with the Declaration of Helsinki and registered at germanctr.de (DRKS00005617). The current study was approved by the Ethics Commission of the Medical Center—University of Freiburg: EK-FR 105/13_130795.

All patients aged 18 years and above that meet the following criteria are eligible to take part in the German Cartilage Registry:

- Conservative or surgical treatment of cartilage defects of the knee, ankle or hip joint at a participating site.
- Signed written informed consent.
- Possession of a personal e-mail address.

In the present study, data of 4986 patients for treatment of cartilage defects of the knee were analyzed. 2445 patients had their main index cartilage defect in the femorotibial compartment. Index cartilage defects in the patellofemoral compartment were not analyzed.

Data collection is performed using a web-based RDE System “RDE-Light” which was developed by the Clinical Trials Unit (Freiburg) as an electronic data entry interface and data management system for clinical studies and other projects in clinical research. Data are collected paperless and directly on site via an internet browser. Forms are based on HTML- and PDF-format. RDE-Light is available in various languages and validated according to GAMP 5. Furthermore, it fulfills all requirements of Good Clinical Practice (GCP). Established security standards like cryptographic security protocols (SSL/TLS), user authentication protocols and authorisation concepts are applied.

After the patient signed the written informed consent the investigator is allowed to register the patients to the database. Patient and defect-specific parameters are reported by the treating physician at the time of surgery while clinical course is evaluated prior to surgery and 6-, 12-, 24-, 36-, 60- and 120-months after surgery. At these time points, the patients’ opinion about their knee function is assessed with standardized instruments (KOOS and IKDC score). Furthermore patient satisfaction, revision surgeries and surgical complication are evaluated with self-administered tools. In this study only baseline data about cartilage treatment procedures and concomitant surgeries on comorbidities like axis deviation, instability or meniscus deficiency according to defect size or genesis of the chondral lesion are presented.

The German Cartilage Registry is supported by a grant from the “Oscar-Helene-Stiftung” and the “Arthrosehilfe e.V.”.

### Statistical analysis

All statistical analyses and descriptive evaluation were performed using SPSS version 25.0 (Chicago, IL, USA). Continuous values are presented as mean (95% CI, range).

## Results

From the initiation in 2013 until August 2019 data of 4968 patients were recorded in the German Cartilage Registry. These patients presented with 2690 cartilage defects in the femorotibial compartment with 2445 patients having the main index cartilage defect in this compartment. The mean age of all patients was 39 years, 64% were male, 36% female. 24% were smokers, 5% ex-smokers and 71% non-smokers. The mean Body Mass Index (BMI) was 27. On average the patient were symptomatic regarding knee pain for 20 months before surgery.

72.6% of all defects in the femorotibial compartment were located at the medial femoral condyle, 20.5% at the lateral condyle, 3.1% at the medial and 3.7% at the lateral tibial plateau. (Table 1).

**Table 1:** Patients’ characteristics

Patients	4968
Patients with main index cartilage lesions in the femorotibial joint	2445
Average age	39 years
Male/female	64%/36%
Smoker yes/ex smoker/no	24%/5%/71%
Average BMI	27
Cartilage defect localization	
Medial femoral condyle	72.6%
Lateral femoral condyle	20.5%
Medial tibial plateau	3.1%
Lateral tibial plateau	3.7%
Mean cartilage lesion size	355 mm^2^

**Fig. 1:**
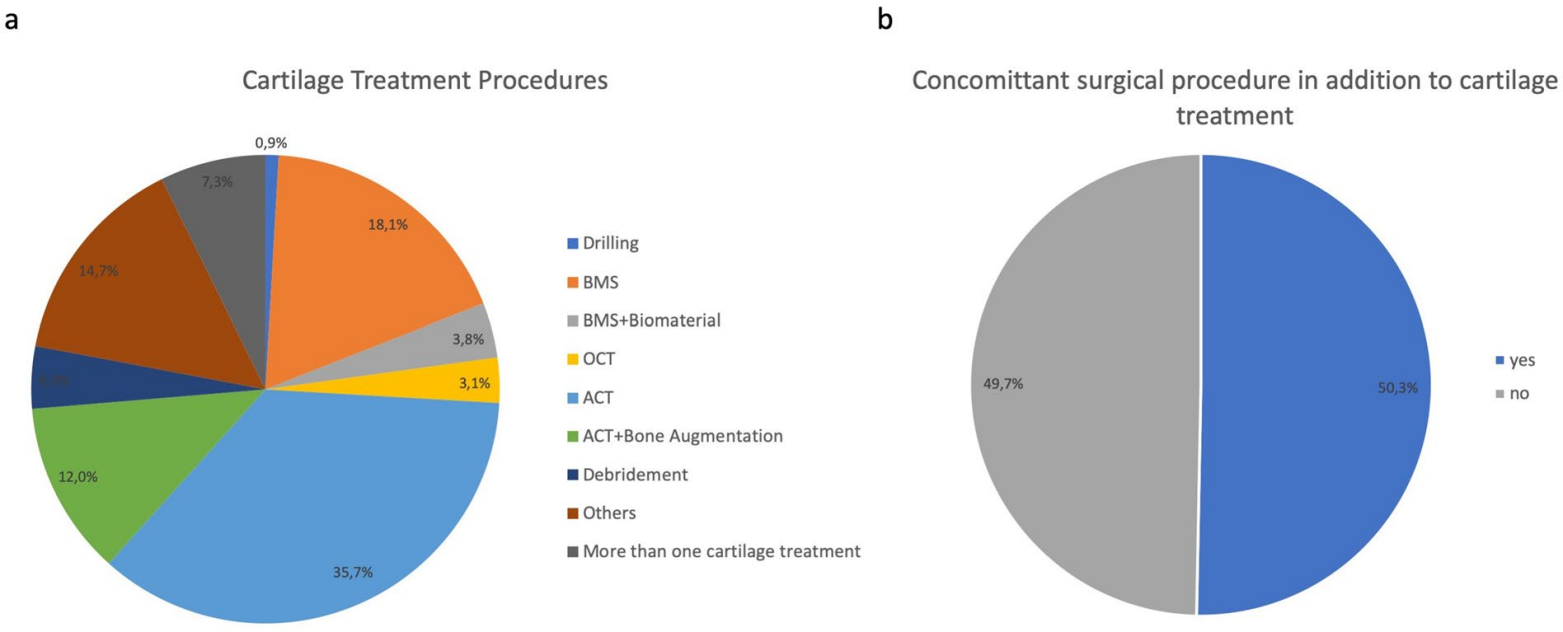
Distribution of the different applied regenerative cartilage repair procedures in all patients with cartilage defects in the femorotibial compartment of the knee (*n* = 2445) (**a**); 50.3% of these patients received an additional surgical procedure to address comorbidities (**b**)

The index cartilage defects of these patients were treated by bone marrow stimulation (BMS) techniques in 18.1% of the cases while ACT was performed in 35.7%. The distribution of other techniques like drilling, OCT, autologous matrix-guided bone marrow stimulation techniques (M-BMS) etc. can be seen in Fig. 1a.

Of the 2445 patients with the main index cartilage lesion in the femorotibial compartment of the knee 1230 (50.3%) were treated with concomitant surgical procedures, while 1215 patients (49.7%) received cartilage repair without additional surgery (Fig. 1b).

**Fig. 2:**
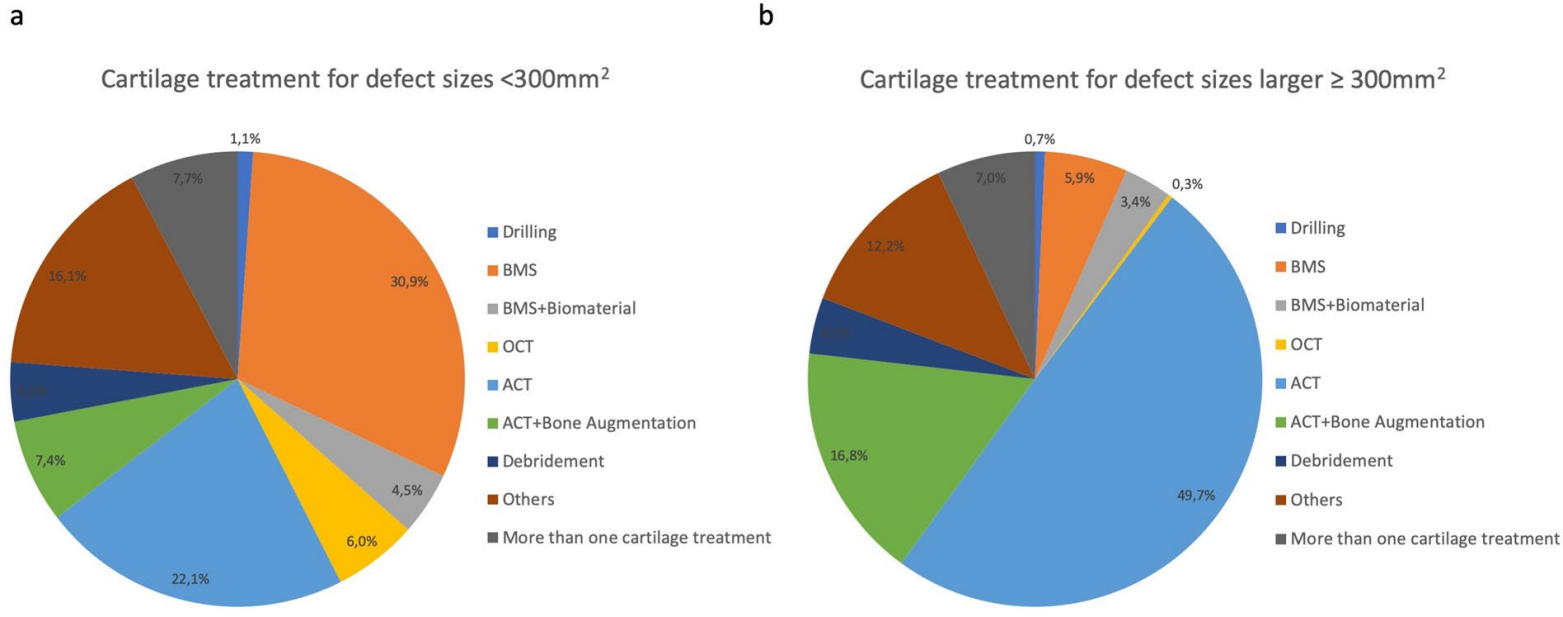
Distribution of different cartilage repair procedures in the femorotibial compartment according defect size < 300 mm^2^ (**a**) and ≥ 300 mm^2^ (**b**)

The mean defect size treated was 355 mm^2^. Regarding cartilage defects smaller than 300 mm^2^ (49.9% of the patients) 51.6% of the patients had concomitant surgeries, while in 48.4% of patients with cartilage lesions larger than 300 mm^2^ (50.1% of all patients) comorbidities were addressed. For defects smaller than 300 mm^2^ (matrix-guided) BMS techniques were used in 35.3% of the cases (ACT: 22.1%) whereas larger defects (> 300 mm^2^) were predominantly treated by ACT with or without bone augmentation (66.6%; (M-) BMS: 9.3%) (Fig. 2).

**Fig. 3:**
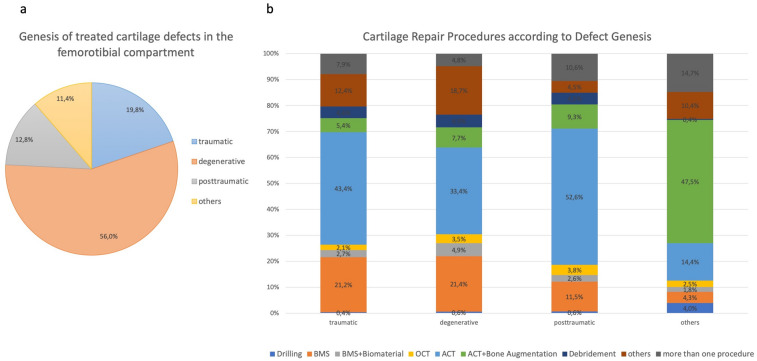
Distribution of different types of defect genesis (**a**) and cartilage repair techniques according to these defect characteristics (**b**)

The genesis of the 2445 treated cartilage defects in the femorotibial compartment were estimated as traumatic in 19%, degenerative in 56.0%, posttraumatic in 12.8% and for other reasons in 11.4% (Fig. 3a). Patients with traumatic and posttraumatic cartilage defects had an average age of 35 years while patients with degenerative lesions were 43 years old on average.

Cartilage defects of all genesis were mainly treated by ACT (traumatic: 43.4%; degenerative 33.4%; posttraumatic 52.6%). In cases with defect causes of other reasons like osteochondritis dissecans the predominant cartilage treatment technique was ACT combined with bone augmentation (47.5%). BMS was the treatment option in 21.2% for traumatic, 21.4% for degenerative and 11.5% for posttraumatic cartilage lesions (Fig. 3b). Comparing BMS vs. ACT a defect treated with BMS was more often degenerative (65.9% vs. 52.3%) but less frequent posttraumatic (8.2% vs. 18.9%). The proportion of traumatic defects was nearly equal in BMS (23.2%) and ACT (24.1%) treated defects.

**Fig. 4:**
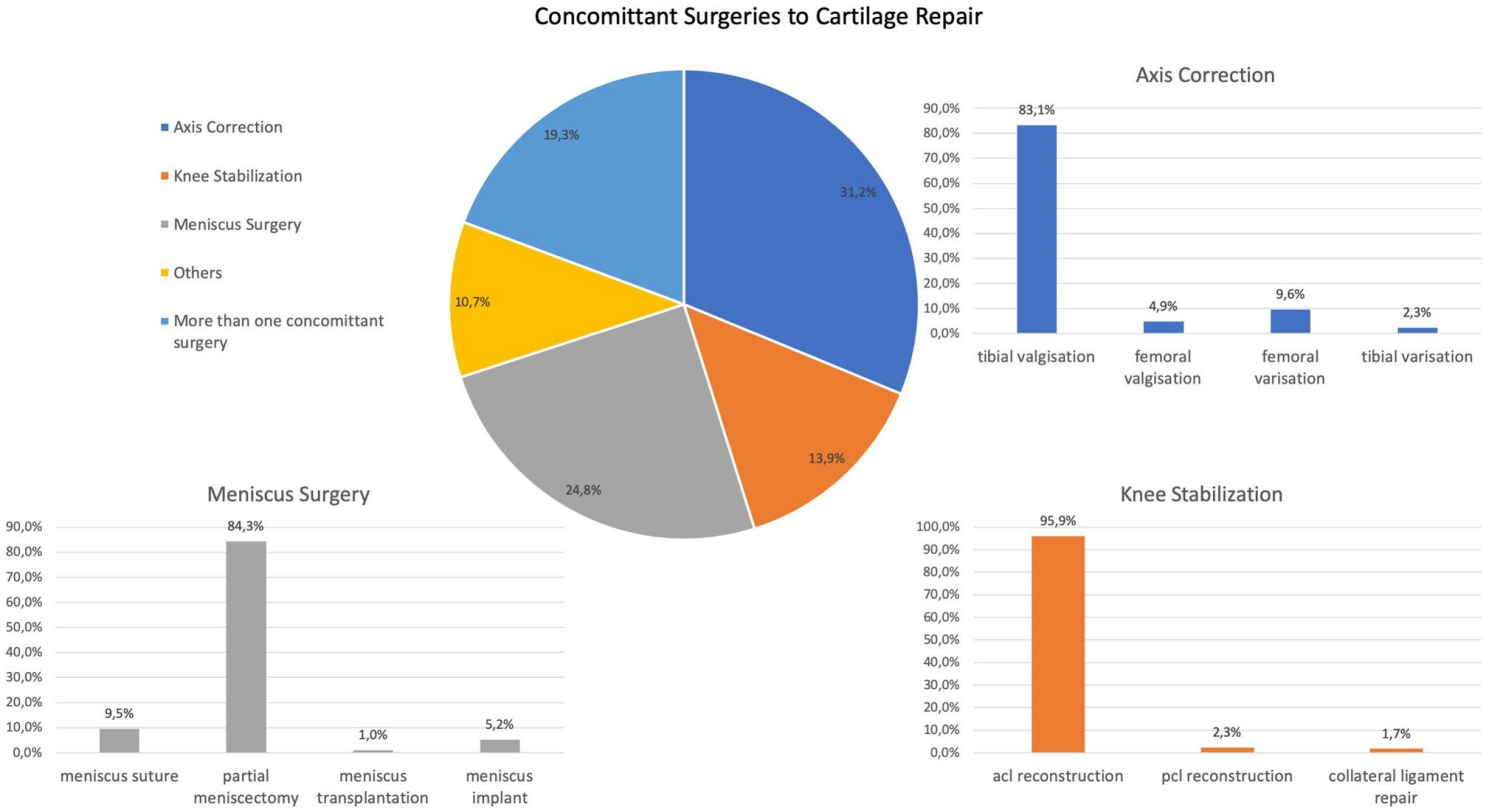
Distribution of different concomitant surgery types in addition to regenerative cartilage repair procedures. Tibial valgisation was the predominant procedure for axis correction and acl reconstruction for concomitant knee stabilization while in additional meniscus surgery most of the patients received a partial meniscectomy

Concomitant surgeries can be separated into mainly three groups: axis correction (performed in 31.2% of patients receiving concomitant surgery), knee stabilization (13.9%) and meniscus surgery (24.8%). 10.7% of patients had other concomitant surgeries, 19.3% of patients received more than one additional procedure (Fig. 4).

The most frequent concomitant surgeries in combination with cartilage treatment were tibial valgisation (*n* = 320, 26.0%), ACL reconstruction (*n* = 165, 13.4%) and partial meniscectomy (*n* = 257, 20.9%) (Fig. 4). The average age of patients who received tibial valgisation was 43 years, while cartilage repair patients treated additionally with ACL reconstruction were 36 years old. Patients for concomitant partial meniscal resection had an average age of 48 years.

**Fig. 5:**
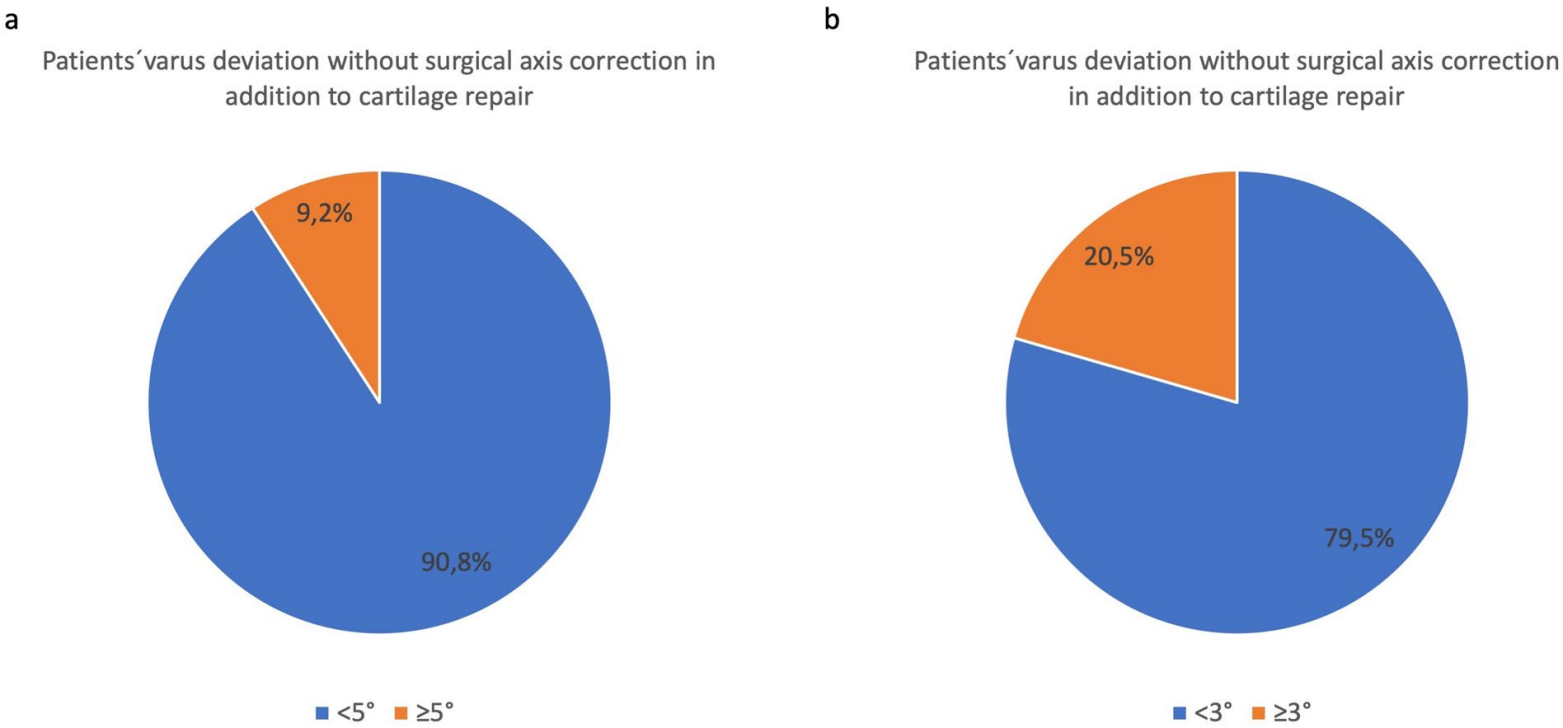
Patients without concomitant surgical axis correction in addition to cartilage repair with more or less than 5° (**a**) or 3° (**b**) varus deviation. 9.2% of patients with a varus deviation of more than 5° and 20.5% of patients with a varus deviation of more than 3° didn’t receive an axis correction together with regenerative cartilage repair procedures

Regarding alignment correction procedures were predominantly performed at the proximal tibia (85.5%) compared to the distal femur (14.5%). Valgisation was more frequently performed at the tibia (94.4%) compared to varisation, which was predominantly corrected at the femur (80.4%). The most frequent axis correction performed together with cartilage repair was tibial valgisation (83.1%) followed by femoral varisation (9.6%). In general, 66.5% of all patients treated for cartilage repair in the femorotibial compartment had preoperative long leg standing X-rays while in 33.5% of the cases no preoperative radiological axis analysis was performed. 97.6% of the patients who received surgical axis deformity correction had long leg standing X-rays for preoperative planning. The average axis deviation was 3.56° of varus. In patients that received no additional axis correction to their cartilage repair procedure 90.8% had less than 5° and 79.5% less than 3° of deviation of their weigh-bearing axis, meaning that 9.2% of patients with an axis deviation of more than 5° and 20.5% with a deviation of more than 3° received no surgical treatment for correction of malalignment in addition to their cartilage repair (Fig. 5).

Concerning knee stabilization surgeries, the most frequent procedure was ACL reconstruction (95.9%) compared to PCL (2.3%)- or collateral ligament reconstruction (1.8%) (Fig. 4).

During cartilage repair procedures surgeons estimated the meniscus status as intact in 53.6%, less than one third resected in 28.1% and more than one third resected in 12.5%. The predominant meniscus procedure in addition to cartilage treatment was partial meniscectomy (84.3%) compared to meniscal suture (9.5%), meniscal allograft transplantation (1.0%) or meniscal supplementation by implants (5.2%) (Fig. 4).

**Fig. 6:**
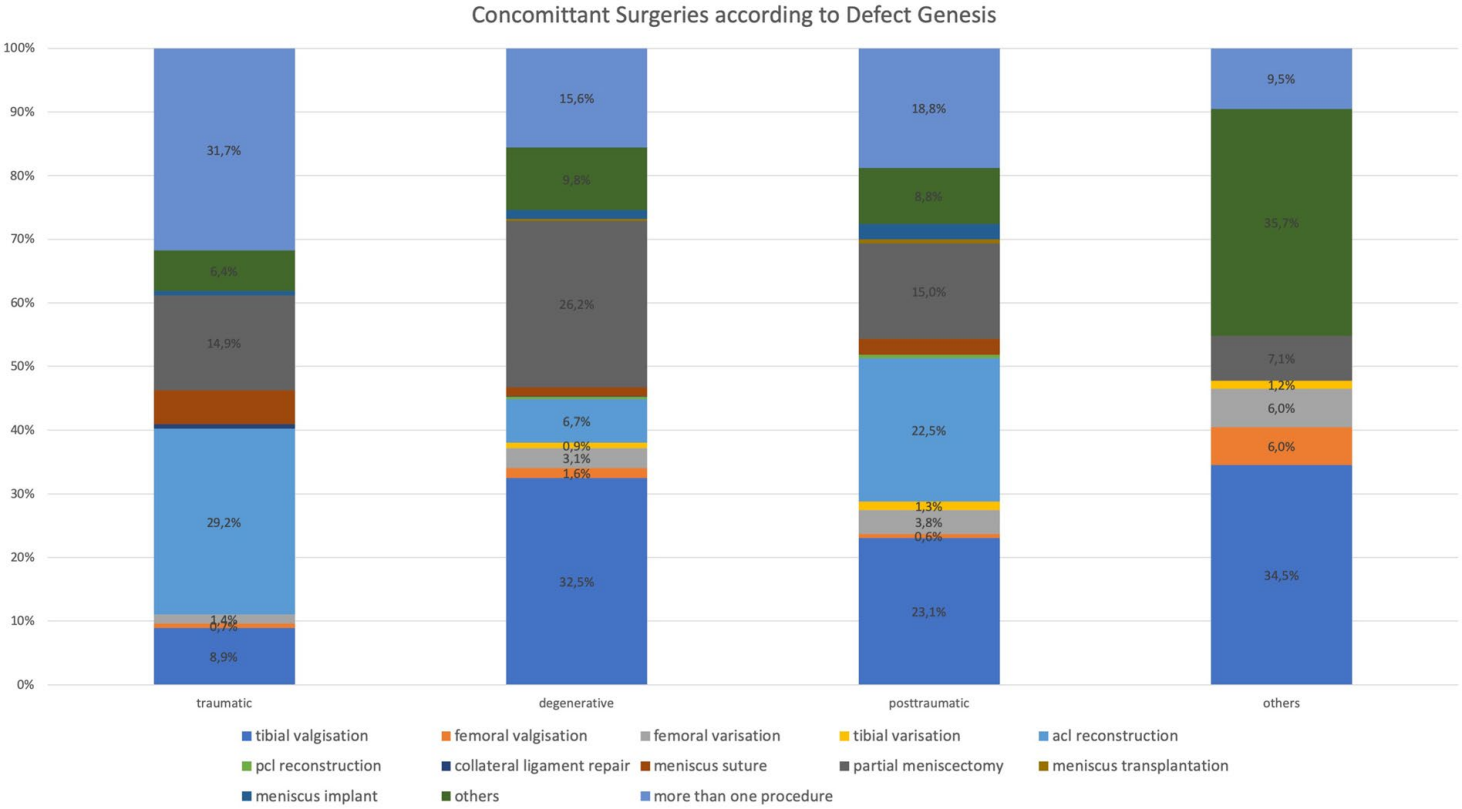
Distribution of different types of concomitant surgery procedures according to the defect genesis. In traumatic and posttraumatic cartilage defects the predominant additional surgery was acl reconstruction while in degenerative cases 32.5% of the patients received a tibial valgisation and 26.2% a partial meniscectomy

The distribution of the types of concomitant surgeries was different related to the genesis of the cartilage defects. In traumatic cartilage lesions the predominant additional procedure was ACL reconstruction (29.2%), in degenerative cartilage lesions only 6.7% received an additional ACL reconstruction. In 31.7% more than one concomitant surgery was necessary to treat traumatic cartilage lesions of the knee. In posttraumatic situations cartilage defects were additionally treated with ACL reconstruction in 22.5%. In 18.8% of the patients more than one additional concomitant surgery was necessary. If an additional treatment of cartilage defect comorbidities was necessary in degenerative situations, the predominant procedure was the correction of the alignment (38.0%) in comparison to traumatic or posttraumatic situations, where 11.0% or 28.8% received an axis correction. In 15.6% of the patients with degenerative cartilage defects more than one concomitant surgery was performed. Partial meniscus resection was needed in addition to the cartilage treatment in 14.9% of traumatic defects, 26.2% of degenerative defects and 15.0% in posttraumatic defects. The most meniscus reconstructions by suture (5.3%) were performed in traumatic situations compared to other types of cartilage defect genesis (degenerative: 1.4%, posttraumatic: 2.5%) (Fig. 6).

## Discussion

This article shows baseline data of the German Cartilage Registry (KnorpelRegister DGOU) regarding concomitant surgeries performed in addition to cartilage treatment. Since the initiation in 2013 4968 patients with 2445 index main cartilage defects in the femorotibial compartment were recorded in the register. Data shows that 50.3% of the patients received additional surgical treatment of concomitant pathologies of the knee indicating that many surgeons esteem cartilage treatment is a whole joint therapy. Increasing numbers of publications show the importance of correction of all concomitant pathologies for a successful regenerative management of cartilage lesions [16, 17, 11]. According to the literature the main addressed comorbidities were malalignment, meniscus deficiency and ligamentous instability.

Varus or valgus malalignment is the major contributing factor to tibiofemoral compartment overload and should be corrected when a cartilage repair procedure is considered to address a chondral lesion in an overloaded compartment [18]. The goal is to restore normal load distribution in the affected compartment to allow cartilage regeneration adjusted to physiologic loads. In this study 385 patients were treated with an osteotomy for malalignment correction of the knee in addition to a cartilage repair procedure in the tibiofemoral joint resembling a portion of 15.7% of all cartilage treated patients and 31.3% of all patients with surgically addressed comorbidities. Varus deviations of the knee are more common and mostly originate in the proximal tibia while most of the valgus deviations derive from the distal femur [19]. The baseline data of the German Cartilage Registry show that the predominant localization for axis correction is the tibia (85.5%) compared to the femur (14.5%). As the most frequent localization of cartilage defects was the medial femoral condyle (72.6%) compared to the lateral condyle (20.5%) the predominant axis correction procedure was tibial valgisation (83.1%) followed by femoral varisation (9.6%).

In case of a varus malalignment, a valgus opening wedge high tibial osteotomy (HTO) combined with a complete MCL release, leads to a significant decrease of pressure and decompression of the medial joint compartment [20]. Kumagai et al. detected cartilage regeneration even in cases of degenerated articular cartilage after opening wedge valgus HTO which was also positively affected by lower BMI [21].

For a correct operative cartilage repair it is mandatory that a potential malalignment is detected by long leg standing X-rays [22, 23] to conceive a treatment strategy and to identify patients who should be indicated for an additional correction of the malalignment. The question arises when a correction osteotomy should be additionally performed and to what extent, to achieve optimal load distribution in the knee. Hohloch et al. analyzed that in combination with an ACI, HTO showed significantly better results regarding Lysholm score and pain VAS, when the postoperative weight-bearing line was between 50 and 55% of the tibial plateau width, compared to a weight-bearing line placed at > 60% [24]. These data show that the weight-bearing axis after correction osteotomy around the knee in combination with regenerative cartilage treatment should be aligned neutral or only in a very mild valgus. In this study 66.5% of all patients treated for cartilage repair in the femorotibial compartment had preoperative long leg standing X-rays while in 33.5% no preoperative radiological axis analysis was performed. Perhaps the relatively high amount of traumatic cases with nearly 20% is an explanation for the high number of preoperatively not analyzed leg axis. However Krych et al. analyzed the mode of failure of primary procedures in consecutive cases in cartilage repair surgery and detected non-addressed malalignment with 56% as the most frequent reasons for failure [25]. Strong efforts must be made in the future to increase the number of preoperative radiological axis evaluation up to 100% before cartilage repair in the femorotibial compartment, where preoperative long leg standing X-ray should be considered as mandatory. At least 97.6% of the patients in this study who received surgical axis deformity correction had long leg standing X-rays for preoperative planning.

Faber et al. found a significant higher postoperative KOOS score, higher satisfaction rate and lower pain levels in patients receiving cartilage repair and varus malalignment with concomitant HTO compared to patients with no additional axis correction after 3 years [26]. Steadman et al. already claimed that additional alignment correction in combination with microfracturing is an effective method for increasing functional outcome and pain relief in patients with cartilage lesions and varus deformities > 5°. While there is a clear general consensus, that varus malalignment > 5° should be addressed by osteotomy when cartilage repair is performed, a European survey revealed, that orthopedic surgeons consider even less than 5° of varus as relevant and recommend to start axis correction for deformities from > 3° [27], which is already implemented in Germany where in an earlier analysis of the German Cartilage Registry by Faber et al. it could be shown that starting from 3° varus the majority of patients received a concomitant osteotomy [16]. Bode et al. compared the outcome after ACI of patients with a varus deformity of 1°–5° with or without an HTO. He found that in this group of patients, HTO leads to a reduced rate of reinterventions and longer survival rate of the regenerative cartilage procedure [28]. In this study only 9.2% of patients with an axis deviation of more than 5° and 20.5% with a deviation of more than 3° received no surgical treatment for correction of malalignment in addition to their cartilage repair. This shows a trend towards correction of malalignment also in cases with smaller axis deviations but the awareness of the correct load distribution in the joint as a key factor for a successful of regenerative cartilage repair can be improved.

Another important factor that contributes to the regenerative potential of cartilage lesions after treatment is the stability of the knee joint. It has been shown that knee ligament insufficiency is linked to an increased risk of development of osteoarthritis over time [29]. Murrell et al. showed that ACL instability contributes to a significant increase in size of cartilage lesions over time. The authors evaluated patients 2 months and 2 years after ACL rupture prior to stabilization and found a six times larger loss of cartilage in patients with longer standing ACL insufficiency [30]. A more recent study by Michalitsis et al. showed similar results. The authors evaluated that the odds of development of a high-grade cartilage lesion in an ACL-deficient knee reconstructed more than 12 months from time from injury are 12.5 higher when compared with knees that underwent ACL reconstruction prior to 12 months after knee injury [31]. In their review, Mehl et al. showed that chronic instability in ACL-deficient knees is associated with a significant increase in medial meniscal injuries after 6 months followed by a significant increase of cartilage lesions after 12 months [32]. These data indicate the importance of knee stability for the cartilage and for the regenerative potential after cartilage treatment to minimize the risk of subsequent failure. In this study 165 ACL reconstruction procedures were performed in 1230 patients that resemble a rate of 13.4% of all concomitant surgeries. Especially in addition to treatment of traumatic cartilage lesions ACL reconstruction was the predominant concomitant surgery (40.4%). Pike et al. saw improved pain and knee function at a long-term follow up, 8 years after combined ACL reconstruction and ACI. In these cases of combined comorbidities, such as instability and malalignment, a tibial osteotomy combining the coronal correction with a slope adaptation for ACL instability can be considered [33]. Non-addressed instability remains one of the most frequent reasons for failed cartilage treatment in the knee [25]. However, significant improvement regarding the surgeons’ awareness of the crucial interaction between stability and cartilage regeneration can be seen in this study.

Another important factor for joint integrity is the status of the meniscus, as it shows a strong interaction between cartilage and also knee stability. The combination of an ACL insufficiency and meniscus deficiency resulted in an 18 times higher cartilage loss 2 years after the ACL injury compared to immediately stabilized knees [30] due to the meniscus’ function as a secondary restraint to anterior tibial translation [34]. Other functions of the meniscus are lubrication, proprioception, shock absorption and load transmission [35]. Biomechanical studies analyzed that the resection of only 15–34% of the meniscus leads to an increase of pressure on the surrounding native cartilage of 350% [36]. After a meniscus tear, cartilage degeneration usually starts at the corresponding location [37]. Otherwise, meniscal alterations like disorganization of the collagen framework, calcification or decrease of mechanical resistance of the meniscus, correlates with the location and the degree of cartilage degeneration [38]. Consecutively partial meniscectomy can be seen as a prearthritic lesion that changes the knee´s integrity and leads to an early onset of osteoarthritis [39]. Although knowing the risk for the onset of osteoarthritis after meniscectomy, the majority of meniscus tears are still treated with partial meniscectomy. During the cartilage repair procedures in this study surgeons estimated the meniscus status as intact in 53.6%, less than one third resected in 28.1% and more than one third resected in 12.5%. According to the increasing knowledge concerning the biology and function of the meniscus, there is a consensus to preserve as much meniscus tissue as possible in the treatment of meniscus injuries, especially in the case of a concomitant cartilage defect [40]. Westin-Barber et al. showed that a successful repair of meniscus tears in the red-white zone indicating an intrinsic healing capacity of meniscal tissue in this critical area [41]. In their meta-analysis Xu et al. show a strong correlation between the amount of restored meniscus tissue and the improvement of functional outcome and prevention of osteoarthritis [42]. In this registry data analysis 305 patients were treated for meniscal lesions which resembles a rate of 24.7% of all patients with concomitant surgeries. However, the predominant meniscus surgery still was the partial meniscectomy (84.3%). The rate of meniscal reconstruction by suture was highest in patients with traumatic cartilage lesions (5.3%).

Interestingly the overall predominant cartilage repair procedure in this study is ACT in even more cases than the even easier performable bone marrow stimulation techniques. The cartilage defect treatment rate is 47.7% for ACT (with or without bone augmentation) compared to only 21.9% bone marrow stimulating techniques (with or without a biomaterial). One reason might be the fact that most of the surgeons that initiated the registry are specialized in the field of regenerative cartilage treatment. Patients might be referred to them after initially failed treatment or with loss of time. Interestingly the surgeons of this study assessed 56.0% of the treated cartilage defects as degenerative, while the genesis of 19.8% was estimated as traumatic and of 12.8% as posttraumatic. The fact that the specialists in cartilage repair treat defects with all these different geneses indicates that degenerative cartilage lesions are no longer a contraindication for a regenerative treatment. Angele et al. showed that also degenerative cartilage lesions in an early OA situation can be treated regeneratively by ACT with a successful clinical outcome [4]. Patients must be informed about the higher failure rate after treatment of degenerative cartilage lesions. The distribution of the concomitant surgeries was different according to the genesis of the defect. While the predominant additional surgery in case of a traumatic cartilage lesion was an ACL reconstruction, the most frequent concomitant surgery for degenerative lesions was the tibial valgisation. By such a whole joint approach with a detailed preoperative analysis and respective concomitant surgeries experienced surgeons are able to push the limits and find indications for regenerative cartilage treatment options also in case of degenerative or early OA situations [43].

An appropriate documentation and analysis are important for such a process. This study gives important baseline information and preliminary data on regenerative cartilage treatment and concomitant surgeries. Follow up data will show which patients and which type of cartilage defect profits the most from regenerative treatment options and additional surgical interventions. Growing interest in the registry and more participating surgeons from different fields (hospital, outpatient clinics) will give a more and more detailed view on the reality of regenerative cartilage treatment and its outcome in the future.

## Conclusion

This baseline analysis of the German Cartilage Registry (KnorpelRegister DGOU) of nearly 5000 patients shows that 50.4% of the patients received a concomitant surgery in addition to a regenerative treatment procedure of cartilage lesions in the femorotibial compartment. The most common comorbidities were axis deviation, knee instability and meniscus deficiency. Data showed that predominant additional surgical treatment included tibial valgisation, ACL reconstruction and partial meniscectomy. The frequency of the several additional surgeries were different regarding to the genesis of the defect with tibial valgisation being the predominant concomitant treatment for degenerative lesions and ACL reconstruction in cases of traumatic chondral defects. Surgeons really seem to analyze the character of a cartilage lesion and try to address all comorbidities as a whole joint therapy. Follow up evaluation from this starting point will enable to analyze the clinical outcome and the importance of treatment of comorbidities together with regenerative cartilage therapy.

## References

[1] Widuchowski W, Widuchowski J, Trzaska T (2007). Articular cartilage defects: study of 25,124 knee arthroscopies. Knee.

[2] Buckwalter JA, Lane NE (1997). Athletics and osteoarthritis. Am J Sports Med.

[3] Shapiro F, Koide S, Glimcher MJ (1993). Cell origin and differentiation in the repair of full-thickness defects of articular cartilage. J Bone Jt Surg.

[4] Angele P, Fritz J, Albrecht D, Koh J, Zellner J (2015). Defect type, localization and marker gene expression determines early adverse events of matrix-associated autologous chondrocyte implantation. Injury.

[5] Brittberg M, Lindahl A, Nilsson A, Ohlsson C, Isaksson O, Peterson L (1994). Treatment of deep cartilage defects in the knee with autologous chondrocyte transplantation. N Engl J Med.

[6] Saris DB, Vanlauwe J, Victor J (2008). Characterized chondrocyte implantation results in better structural repair when treating symptomatic cartilage defects of the knee in a randomized controlled trial versus microfracture. Am J Sports Med.

[7] Zellner J, Angele P, Zeman F, Kujat R, Nerlich M (2013). Is the transplant quality at the time of surgery adequate for matrix-guided autologous cartilage transplantation? A pilot study. Clin Orthop Relat Res.

[8] Khan IM, Gilbert SJ, Singhrao SK, Duance VC, Archer CW (2008). Cartilage integration: evaluation of the reasons for failure of integration during cartilage repair. A review. Eur Cells Mater.

[9] Niemeyer P, Pestka JM, Kreuz PC (2008). Characteristic complications after autologous chondrocyte implantation for cartilage defects of the knee joint. Am J Sports Med.

[10] Niemeyer P, Albrecht D, Andereya S (2016). Autologous chondrocyte implantation (ACI) for cartilage defects of the knee: a guideline by the working group "clinical tissue regeneration" of the german society of orthopaedics and trauma (DGOU). Knee.

[11] Lattermann C, Luckett MR (2011). Staging and comorbidities. J Knee Surg.

[12] Kolling C, Simmen BR, Labek G, Goldhahn J (2007). Key factors for a successful national arthroplasty register. J Bone Jt Surg.

[13] Granan LP, Bahr R, Lie SA, Engebretsen L (2009). Timing of anterior cruciate ligament reconstructive surgery and risk of cartilage lesions and meniscal tears: a cohort study based on the Norwegian national knee ligament registry. Am J Sports Med.

[14] Niemeyer P, Schweigler K, Grotejohann B (2015). The German cartilage registry (KnorpelRegister DGOU) for evaluation of surgical treatment for cartilage defects: experience after six months including first demographic data. Zeitschrift fur Orthopadie und Unfallchirurgie.

[15] Spahn G, Fritz J, Albrecht D, Hofmann GO, Niemeyer P (2016). Characteristics and associated factors of Klee cartilage lesions: preliminary baseline-data of more than 1000 patients from the German cartilage registry (KnorpelRegister DGOU). Arch Orthop Trauma Surg.

[16] Faber S, Zellner J, Angele P (2020). Decision making for concomitant high tibial osteotomy (HTO) in cartilage repair patients based on a nationwide cohort study of 4968 patients. Arch Orthop Trauma Surg.

[17] Faber S, Zinser W, Angele P (2020). Does gender influence outcome in cartilage repair surgery? An analysis of 4,968 consecutive patients from the German cartilage registry (Knorpel Register DGOU). Cartilage.

[18] Brittberg M, Tallheden T, Sjogren-Jansson B, Lindahl A, Peterson L (2001). Autologous chondrocytes used for articular cartilage repair: an update. Clin Orthop Relat Res.

[19] Paley D, Herzenberg JE, Tetsworth K, McKie J, Bhave A (1994). Deformity planning for frontal and sagittal plane corrective osteotomies. Orthop Clin North Am.

[20] Agneskirchner JD, Hurschler C, Wrann CD, Lobenhoffer P (2007). The effects of valgus medial opening wedge high tibial osteotomy on articular cartilage pressure of the knee: a biomechanical study. Arthroscopy.

[21] Kumagai K, Akamatsu Y, Kobayashi H, Kusayama Y, Koshino T, Saito T (2017). Factors affecting cartilage repair after medial opening-wedge high tibial osteotomy. Knee Surg Sports Traumatol Arthrosc.

[22] Brinkman JM, Lobenhoffer P, Agneskirchner JD, Staubli AE, Wymenga AB, van Heerwaarden RJ (2008). Osteotomies around the knee: patient selection, stability of fixation and bone healing in high tibial osteotomies. J Bone Jt Surg.

[23] Holschen M, Lobenhoffer P (2016). Complications of corrective osteotomies around the knee. Der Orthopade.

[24] Hohloch L, Kim S, Mehl J (2018). Customized post-operative alignment improves clinical outcome following medial open-wedge osteotomy. Knee Surg Sports Traumatol Arthrosc.

[25] Krych AJ, Hevesi M, Desai VS, Camp CL, Stuart MJ, Saris DBF (2018). Learning From failure in cartilage repair surgery: an analysis of the mode of failure of primary procedures in consecutive cases at a tertiary referral center. Orthop J Sports Med.

[26] Faber S, Angele P, Zellner J, Bode G, Hochrein A, Niemeyer P (2020). Comparison of clinical outcome following cartilage repair for patients with underlying varus deformity with or without additional high tibial osteotomy: a propensity score-matched study based on the German cartilage registry (KnorpelRegister DGOU). Cartilage.

[27] Feucht MJ, Minzlaff P, Saier T (2014). Degree of axis correction in valgus high tibial osteotomy: proposal of an individualised approach. Int Orthop.

[28] Bode G, Ogon P, Pestka J (2015). Clinical outcome and return to work following single-stage combined autologous chondrocyte implantation and high tibial osteotomy. Int Orthop.

[29] Lohmander LS, Englund PM, Dahl LL, Roos EM (2007). The long-term consequence of anterior cruciate ligament and meniscus injuries: osteoarthritis. Am J Sports Med.

[30] Murrell GA, Maddali S, Horovitz L, Oakley SP, Warren RF (2001). The effects of time course after anterior cruciate ligament injury in correlation with meniscal and cartilage loss. Am J Sports Med.

[31] Michalitsis S, Vlychou M, Malizos KN, Thriskos P, Hantes ME (2015). Meniscal and articular cartilage lesions in the anterior cruciate ligament-deficient knee: correlation between time from injury and knee scores. Knee Surg Sports Traumatol Arthrosc.

[32] Mehl J, Otto A, Baldino JB (2019). The ACL-deficient knee and the prevalence of meniscus and cartilage lesions: a systematic review and meta-analysis (CRD42017076897). Arch Orthop Trauma Surg.

[33] Feucht MJ, Mauro CS, Brucker PU, Imhoff AB, Hinterwimmer S (2013). The role of the tibial slope in sustaining and treating anterior cruciate ligament injuries. Knee Surg Sports Traumatol Arthrosc.

[34] Allen CR, Wong EK, Livesay GA, Sakane M, Fu FH, Woo SL (2000). Importance of the medial meniscus in the anterior cruciate ligament-deficient knee. J Orthop Res.

[35] Makris EA, Hadidi P, Athanasiou KA (2011). The knee meniscus: structure-function, pathophysiology, current repair techniques, and prospects for regeneration. Biomaterials.

[36] Radin EL, de Lamotte F, Maquet P (1984). Role of the menisci in the distribution of stress in the knee. Clin Orthop Relat Res.

[37] Madry H, Luyten FP, Facchini A (2012). Biological aspects of early osteoarthritis. Knee Surg Sports Traumatol Arthrosc.

[38] Du G, Zhan H, Ding D (2016). Abnormal mechanical loading induces cartilage degeneration by accelerating meniscus hypertrophy and mineralization after ACL injuries in vivo. Am J Sports Med.

[39] McDermott ID, Amis AA (2006). The consequences of meniscectomy. J Bone Jt Surg.

[40] Verdonk R, Madry H, Shabshin N (2016). The role of meniscal tissue in joint protection in early osteoarthritis. Knee Surg Sports Traumatol Arthrosc.

[41] Barber-Westin SD, Noyes FR (2014). Clinical healing rates of meniscus repairs of tears in the central-third (red–white) zone. Arthroscopy.

[42] Xu C, Zhao J (2015). A meta-analysis comparing meniscal repair with meniscectomy in the treatment of meniscal tears: the more meniscus, the better outcome?. Knee Surg Sports Traumatol Arthrosc.

[43] Angele P, Niemeyer P, Steinwachs M (2016). Chondral and osteochondral operative treatment in early osteoarthritis. Knee Surg Sports Traumatol Arthrosc.

